# *In vitro* prion protein conversion suggests risk of bighorn sheep (*Ovis canadensis*) to transmissible spongiform encephalopathies

**DOI:** 10.1186/1746-6148-9-157

**Published:** 2013-08-09

**Authors:** Aaron R Morawski, Christina M Carlson, Haeyoon Chang, Christopher J Johnson

**Affiliations:** 1 Department of Bacteriology, University of Wisconsin, Madison, WI, USA; 2 USGS National Wildlife Health Center, Madison, WI, USA; 3 Program in Cellular and Molecular Biology, University of Wisconsin, Madison, WI, USA; 4 Present address: National Institutes of Health, 9000 Rockville Pike, Bethesda 20892, Maryland, USA

**Keywords:** Bighorn sheep, Scrapie, Chronic wasting disease, Transmissible mink encephalopathy, Species barrier

## Abstract

**Background:**

Transmissible spongiform encephalopathies (TSEs) affect both domestic sheep (scrapie) and captive and free-ranging cervids (chronic wasting disease; CWD). The geographical range of bighorn sheep (*Ovis canadensis*; BHS) overlaps with states or provinces that have contained scrapie-positive sheep or goats and areas with present epizootics of CWD in cervids. No TSEs have been documented in BHS, but the susceptibility of this species to TSEs remains unknown.

**Results:**

We acquired a library of BHS tissues and found no evidence of preexisting TSEs in these animals. The prion protein gene (*Prnp*) in all BHS in our library was identical to scrapie-susceptible domestic sheep (A^136^R^154^Q^171^ genotype). Using an *in vitro* prion protein conversion assay, which has been previously used to assess TSE species barriers and, in our study appears to recollect known species barriers in mice, we assessed the potential transmissibility of TSEs to BHS. As expected based upon *Prnp* genotype, we observed BHS prion protein conversion by classical scrapie agent and evidence for a species barrier between transmissible mink encephalopathy (TME) and BHS. Interestingly, our data suggest that the species barrier of BHS to white-tailed deer or wapiti CWD agents is likely low. We also used protein misfolding cyclic amplification to confirm that CWD, but not TME, can template prion protein misfolding in A^136^R^154^Q^171^ genotype sheep.

**Conclusions:**

Our results indicate the *in vitro* conversion assay used in our study does mimic the species barrier of mice to the TSE agents that we tested. Based on *Prnp* genotype and results from conversion assays, BHS are likely to be susceptible to infection by classical scrapie. Despite mismatches in amino acids thought to modulate prion protein conversion, our data indicate that A^136^R^154^Q^171^ genotype sheep prion protein is misfolded by CWD agent, suggesting that these animals could be susceptible to CWD. Further investigation of TSE transmissibility to BHS, including animal studies, is warranted. The lack of reported TSEs in BHS may be attributable to other host factors or a lack of TSE surveillance in this species.

## Background

Prior to human settlement, bighorn sheep (*Ovis canadensis*; BHS) were widely distributed and abundant in western North America [1]. Overharvest, habitat loss and disease have contributed to population declines and modern-day numbers of BHS are thought to be reduced by more than 90% compared to those during pre-settlement times [1]. More than half of existing BHS populations are the result of restoration efforts and certain subspecies are presently endangered or have gone extinct [2,3]. Disease continues to be a major obstacle to BHS recovery [1]. The transfer of pathogens, such as *Mannheimia haemolytica*, from domestic sheep (*Ovis aires*) to BHS and between BHS herds has caused numerous mortality events and has been modeled under experimental conditions [4-6]. While less well characterized, cervids like white-tailed deer (*Odocoileus virginianus*), mule deer (*O. hemionus*), wapiti (American elk; *Cervus canadensis*) or moose (*Alces alces*) may also share disease with BHS [7].

**Figure 1:**
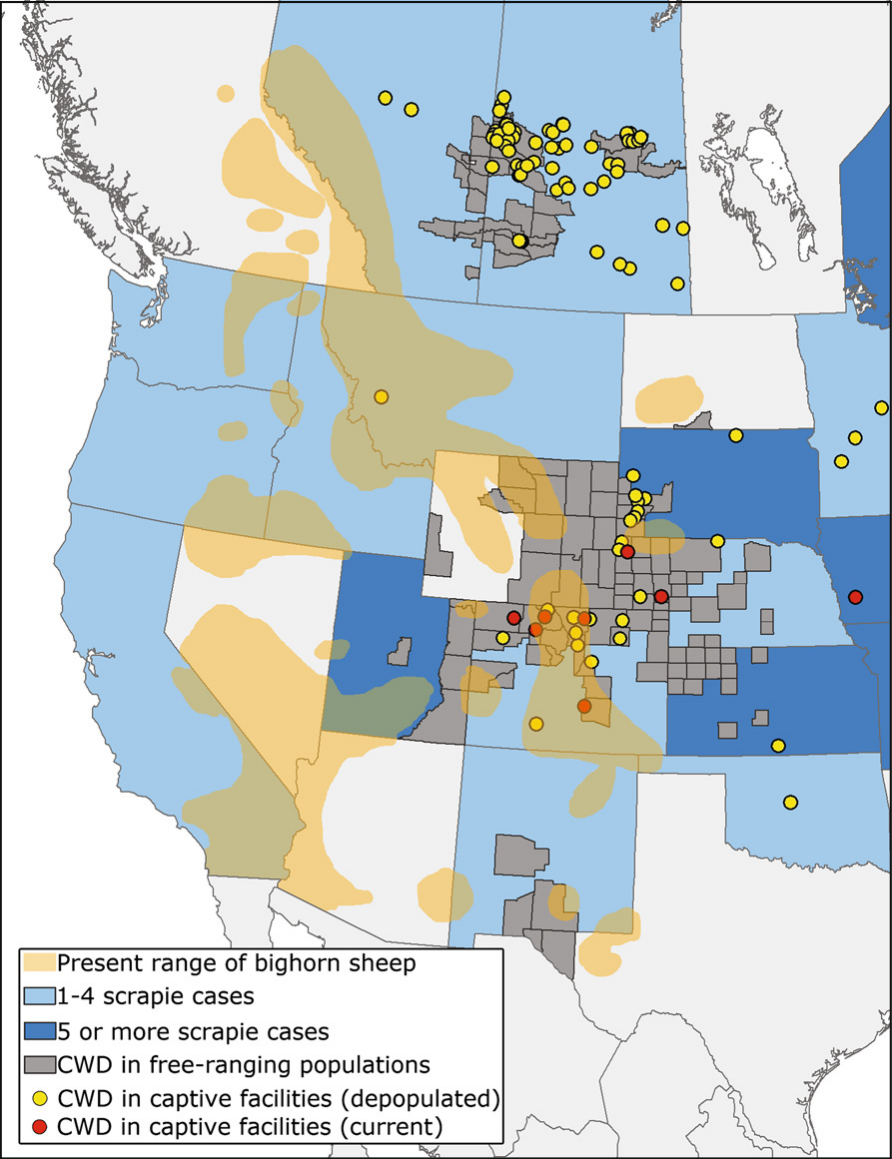
**Overlap of bighorn sheep range with scrapie and chronic wasting disease (CWD).** Orange regions represent the present range of bighorn sheep (data from Grand Slam/Ovis). Blue shading indicates either 1–4 (light blue) or 5 or more (dark blue) cases of scrapie in a state or province since October, 2008 (data from US Department of Agriculture and Scrapie Canada). Grey coloring represents areas where CWD has been identified in free-ranging cervid populations. Yellow or red dots indicate CWD in depopulated or active captive facilities, respectively.

Domestic sheep and free-ranging and farmed cervids are all affected by transmissible spongiform encephalopathies (TSEs, prion diseases) [8]. Scrapie is a TSE of domestic sheep and goats that is endemic in many countries [9]. Chronic wasting disease (CWD) is an emerging prion disease that affects deer, wapiti and moose [10]. Since its identification in 1967, CWD has been detected in captive or free-ranging deer, wapiti or moose populations in at least 22 states, 2 Canadian provinces and South Korea. In captive populations, CWD can reach nearly 100% prevalence [11] and while the upper limit of CWD prevalence in free-ranging populations is not yet known, some portions of Wyoming have recently observed >50% prevalence [12]. Scrapie and CWD infectivity is shed from diseased animals, allowing transmission to naïve hosts [13]. Remarkably, scrapie and CWD infectivity shed into the environment persists for long periods of time (years to decades) and can cause disease when naïve animals are later exposed [14,15]. Direct contact with shed scrapie or CWD agent, as well as environmental sources of infectivity, are both plausible mechanisms of TSE transmission whereby new species, such as BHS, could be exposed to disease. The range of BHS substantially overlaps with states and provinces known to have had recent scrapie outbreaks, areas where CWD is endemic in free-ranging cervids and captive cervid facilities where CWD has been found (Figure 1).

The infectious agent responsible for TSEs is thought to be a misfolded protein, termed a prion. During infection, the normal, host cellular prion protein (PrP^C^) is converted to a misfolded form (PrP^TSE^) that accumulates in the nervous system, is associated with disease and is a primary component of the infectious prion agent [16]. Proteinase K (PK) is used to discriminate between the two conformers of prion protein; PrP^C^ is completely degraded following PK treatment whereas the protease cleaves only the *N*-terminus from PrP^TSE^ and leaves an infectious core intact [17]. The presence of PK-resistant PrP (PrP^res^) in a sample is a common diagnostic indicator of TSE infection [18].

For interspecies TSE transmission, the degree to which host PrP^C^ matches the sequence of the infectious PrP^TSE^ can be a key determinant in whether conversion will take place [19]. While animal challenges remain the most definitive measure of the susceptibility of a species to a TSE, a number of *in vitro* assays have been developed to assess the potential for interspecies TSE transmissions. Such assays typically assess the conversion of host PrP^C^ to a PK-resistant state when seeded by PrP^TSE^. Cell-free conversion assays, protein misfolding cyclic amplification (PMCA) and the conversion efficiency ratio (CER) assay have all been used to assess TSE species barriers [20-23]. In the CER assay, two denatured PrP^C^ substrates are used in conversions [24]. One of the denatured substrates is processed at pH 7.4 and a previous report indicates that only in the absence of a species barrier is the PrP^C^ in the substrate converted to PrP^res^ by PrP^TSE^[23]. The other PrP^C^ substrate is processed at pH 3.5 and can be converted to PrP^res^ following incubation with any species of PrP^TSE^. Comparing PrP^res^ levels in these two substrates provides a measure of the species barrier. Li *et al.* introduced this assay in an effort to predict the species barriers of numerous animals to wapiti CWD [23]. In the present manuscript, we assess the *Prnp* genotype of 28 BHS in a tissue library and find no evidence of TSE in these tissues. We confirm that the CER assay correctly predicts known species barriers of laboratory mice to various TSEs and go on to use this assay to assess PrP^C^ conversion in BHS substrates by domestic sheep classical scrapie, transmissible mink encephalopathy (TME) and CWD.

## Results

### Sequence analysis of BHS

**Figure 2:**
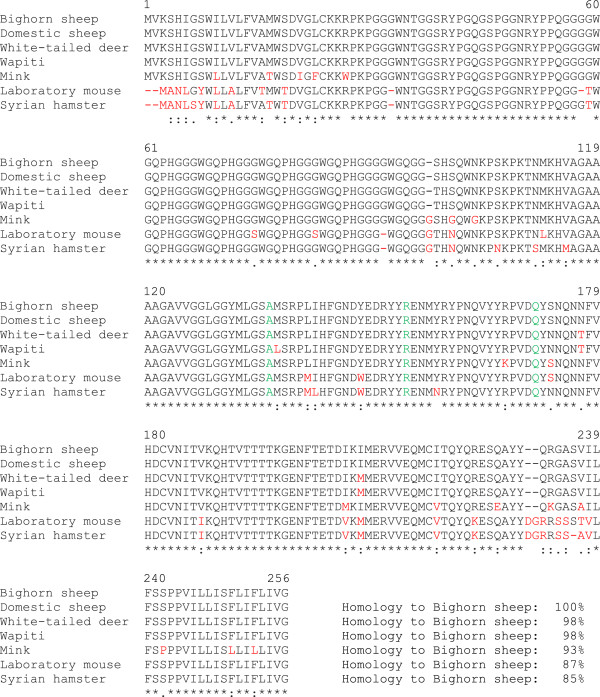
**Prion protein amino acid alignment of bighorn sheep, domestic sheep (A**^**136**^**R**^**154**^**Q**^**171**^**), white-tailed deer, wapiti, mink, laboratory mouse and hamster.** Green letters indicate key amino acids for scrapie susceptibility. Red letters indicate mismatches. Below sequence the alignment, consensus symbols (* identical amino acids, amino acids with strongly similar properties, amino acids with weakly similar properties) are displayed.

We isolated genomic DNA from 28 BHS in our tissue library and sequenced their prion protein gene (*Prnp*). A previous report and entry in GenBank (AAD48030.1) indicated that the prion protein from a group of BHS has the same amino acid sequence as scrapie-susceptible A^136^R^154^Q^171^ genotype domestic sheep [21]. Our results were identical to the previously published report and no amino acid changes were found in any BHS sequenced. An alignment of BHS prion protein with that of other species is shown in Figure 2 and the similarity between BHS, domestic sheep, white-tailed deer and wapiti prion proteins was >95%. The mink, laboratory mouse and Syrian hamster prion proteins are more divergent.

### Screening BHS tissues for TSEs

**Figure 3:**
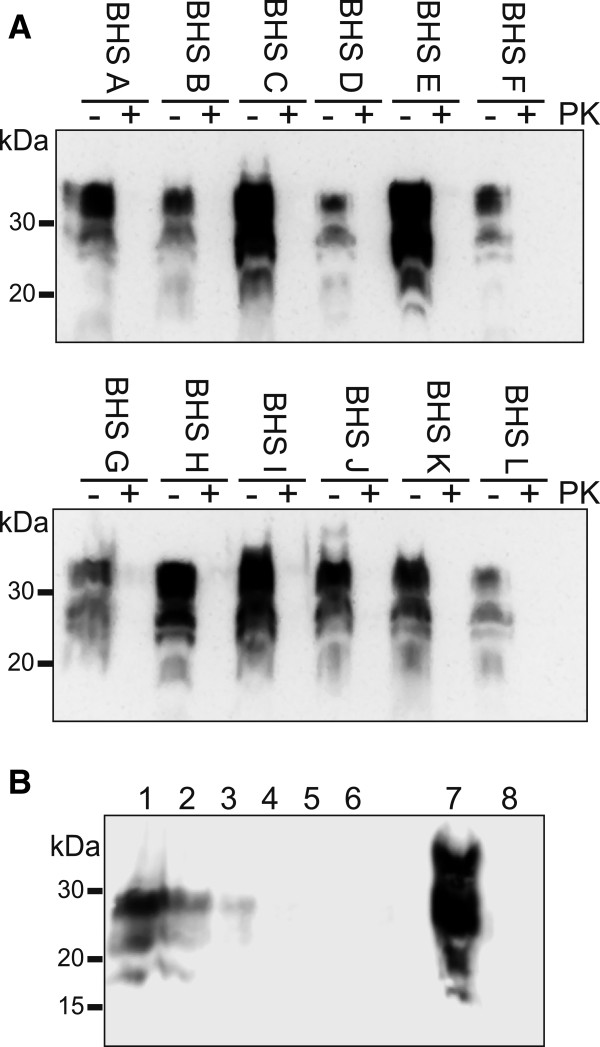
**No evidence of proteinase K (PK)-resistant prion protein in bighorn sheep brain tissues. (A)** Individual bighorn sheep (BHS) were given unique letters to identify them (A-L). The obex region of the medulla from each animal was collected, homogenized to 10% w/v, a 25 *μ*L aliquot of each homogenate incubated in the presence of 50 *μ*g·ml^-1^ of proteinase K (+) or in the absence of PK (−) and then immunoblotted. Samples from the other BHS in our tissue library similarly did not have PK-resistant prion protein. **(B)** A dilution series of classical scrapie brain homogenate was prepared in BHS brain homogenate, incubated in the presence of 50 *μ*g·ml^-1^ of proteinase K and immunoblotted. Scrapie dilution factors were 3, 10, 30, 100, 300 and 1000 (lanes 1–6). Lanes 7 and 8 represent non-proteinase K-treated and proteinase K-treated BHS brain homogenate in the absence of scrapie brain homogenate, respectively. Immunoblots used monoclonal antibody BAR 224.

The sequence identity between domestic sheep and BHS prion proteins allowed us to use reagents tested in domestic sheep for analysis of prion protein in BHS tissues. We tested the BHS in our tissue library for evidence of preexisting TSEs by examining PK-treated brain homogenates for the presence of PrP^res^ by immunoblotting (Figure 3A). Samples of brain were taken from the obex region of the medulla oblongata, homogenized, digested with PK or mock digested and immunoblotted. No PrP^res^ was detected in any BHS sample, suggesting the animals were free of TSE. The PrP^C^ signal observed in the non-PK-digested immunoblot samples was generally abundant (Figure 3A), indicating a lack of proteolysis in our tissue samples and encouraging our use of these tissues as substrates for *in vitro* conversion assays. To test the sensitivity of our immunoblotting procedures, we prepared dilutions of brain homogenate from a domestic sheep that was clinically-ill with classical scrapie in BHS brain homogenate and PK treated the mixtures. We found that we could detect scrapie PrP^res^ out to a dilution factor of 30 (Figure 3B), indicating that PrP^res^ levels in the BHS were below this threshold. As an additional control, we tested if we could detect PrP^Sc^ in BHS brain tissues selected for use in conversion assays following three rounds of serial protein misfolding cyclic amplification. We did not detect any PrP^res^ in any amplified sample (data not presented).

Retropharyngeal lymph nodes are among the first sites where abnormal prion protein can be detected in some preclinical TSEs and testing this tissue is useful in diagnosing domestic sheep scrapie [25]. Despite no evidence of PrP^res^ in our library of BHS brain tissue, we screened retropharyngeal lymph nodes from the 28 BHS using the BioRad TeSeE ELISA test, an assay commonly used to test domestic sheep for scrapie. We found no positive samples (data not shown), consistent with the idea that our BHS tissues do not harbor preexisting TSEs.

### Control conversion efficiency ratio (CER) studies

The species barrier of laboratory mice to most TSEs is well-characterized [26]. For that reason, we began our investigations with the CER assay using mouse PrP^C^ substrates and TSE agents that have been used in previous mouse bioassays. Specifically, we used the RML strain of mouse-passaged scrapie, an agent adapted to passage in mice [27], domestic sheep classical scrapie, an agent that can transmit to mice following a lengthy incubation period [27] and CWD and 263K strain of hamster-passaged scrapie, agents to which mice are minimally or not susceptible [28,29].

**Figure 4:**
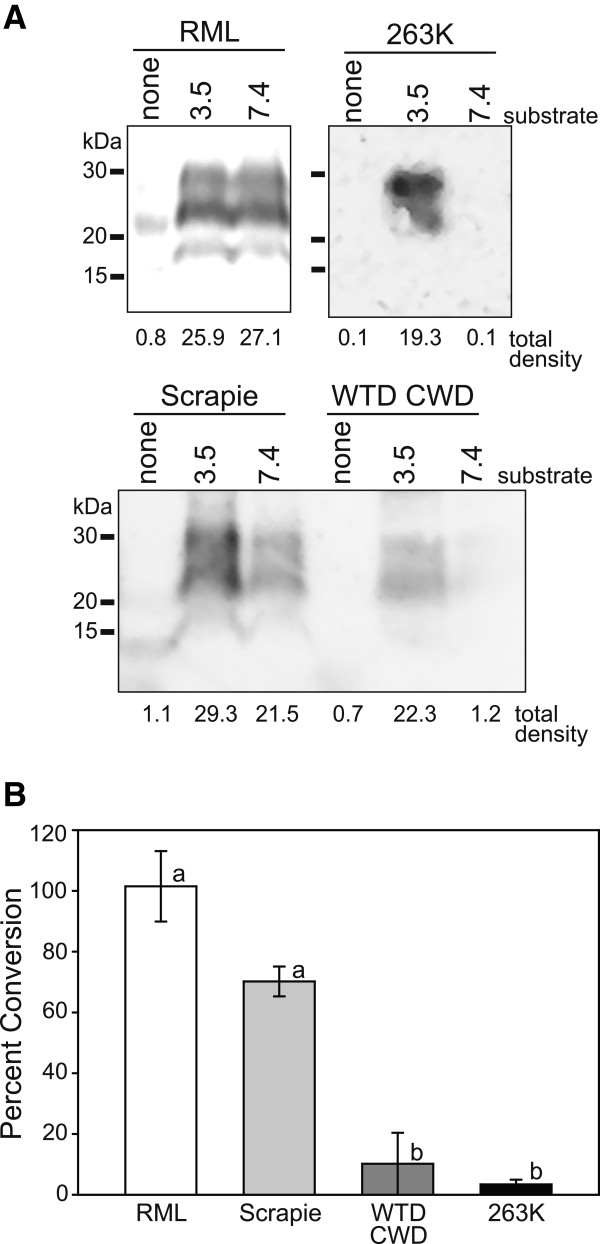
**Conversion efficiency ratio (CER) assay using laboratory mouse substrate. (A)** Mouse CER assay substrate prepared at either pH 7.4 or 3.5 was incubated with RML mouse-adapted scrapie, domestic sheep classical scrapie, white-tailed deer (WTD) chronic wasting disease agent (CWD) or 263K strain of hamster-adapted scrapie. Control samples (labeled “none”) contained only an equal amount of infectious agent and no mouse substrate. Samples were analyzed for the presence of proteinase K-resistant prion protein by immunoblot with monoclonal antibody SAF 83. Raw densitometric values for each sample are displayed below each lane. **(B)** Bar graph indicating the average ratios (+/− standard deviation) between pH 7.4 and 3.5 mouse substrates for each infectious agent based on at least three independent assay runs. Lower-case letters refer to statistically homogeneous subsets (analysis of variance with Tukey-Kramer minimum significance differences method; p < 0.05).

We incubated mouse PrP^C^ substrates with these TSE agents and assessed conversion of PrP^C^ to PrP^res^ (Figure 4A). In mouse PrP^C^ substrate denatured at pH 7.4, these TSE agents produced a range of PrP^res^ levels whereas in the substrate denatured at pH 3.5, PrP^res^ was found in all samples incubated with a TSE agent. The ratios comparing the PrP^res^ levels in the pH 7.4 and pH 3.5 substrates were independently examined at least three times and means +/− SD are shown in Figure 4B. The conversion ratios between pH 7.4 and 3.5 substrates seeded by RML were approximately 100%. Scrapie-induced conversion of the pH 7.4 substrate was approximately 75% of the pH 3.5 substrate and conversion of the pH 7.4 substrate by either CWD or 263K was minimal. A one-way analysis of variance did not find a significant difference between RML and scrapie or CWD and 263K conversion ratios. Conversion ratios of RML and scrapie were, however, significantly different from CWD and 263K.

### Experimental CER studies

With evidence suggesting that PrP^C^ in our BHS brain tissues was intact and present at detectable levels (Figure 3), and that the CER assay can reproduce mouse TSE species barriers (Figure 4), we generated CER assay substrates from BHS brain to assess the conversion of BHS PrP^C^ by TSE agents. We chose to assess domestic classical sheep scrapie because BHS and A^136^R^154^Q^171^ genotype domestic sheep share an identical *Prnp* sequence and selected isolates of CWD because of range overlap. Transmissible mink encephalopathy (TME) agent was used as a control because there are multiple sequence differences between mink and BHS prion proteins (Figure 2) and because a previous study indicated limited transmissibility of TME to domestic sheep (Cheviot breed) with an extended incubation period (mean: 65 mo) and with only about 25% penetrance [29].

**Figure 5:**
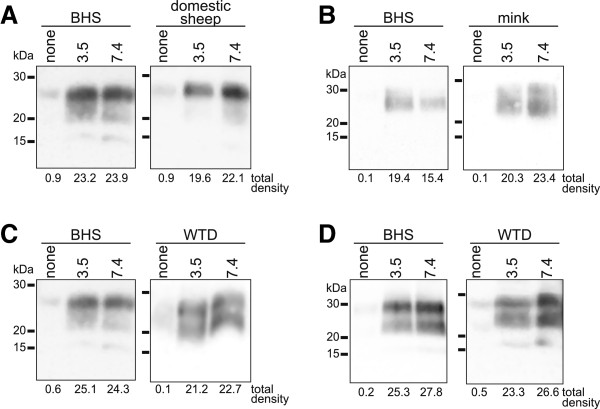
**Conversion efficiency ratio (CER) assay using bighorn sheep (BHS) and native host species substrates.** Immunoblots to assess proteinase K-resistant prion protein in BHS CER assay substrate prepared at either pH 7.4 or 3.5 and incubated with **(A)** domestic sheep classical scrapie, **(B)** transmissible mink encephalopathy (TME) agent, **(C)** white-tailed deer (WTD) chronic wasting disease (CWD) agent or **(D)** wapiti CWD agent. Domestic sheep, mink or white-tailed deer substrates were used as controls in **A**, **B** or **C** &**D**, respectively. Lanes labeled “none” contained an equal amount of the indicated TSE agent but no substrate. Raw densitometric values for each sample are displayed below each lane. Immunoblots used monoclonal antibody BAR 224.

In Figure 5, we assessed conversion of BHS PrP^C^ to PrP^res^ by these various TSE agents. We found that domestic sheep classical scrapie induced PrP^C^ to PrP^res^ conversion in BHS and domestic sheep pH 7.4 substrates, as well as pH 3.5 substrates (Figure 5A). In BHS samples, some PrP^res^ was detectable in pH 7.4 substrate incubated with TME agent, however the amount appeared somewhat diminished compared to the BHS pH 3.5 substrates (Figure 5B). In mink, TME agent caused PrP^C^ to PrP^res^ conversion in both pH 7.4 and 3.5 samples. In BHS samples incubated with white-tailed deer CWD, PrP^res^ was detected in both pH 7.4 and 3.5 substrates (Figure 5C). In Figure 5D, similar results were obtained using an isolate of CWD from a wapiti heterozygous methionine/leucine at position 132 (M/L^132^). Both isolates of CWD caused PrP^C^ to PrP^res^ conversion in white-tailed deer (Figure 5C and D). Control samples of substrates prepared at pH 7.4 and 3.5 for all species were run in the absence of TSE agents and PrP^res^ was not detected (Additional file 1).

**Figure 6:**
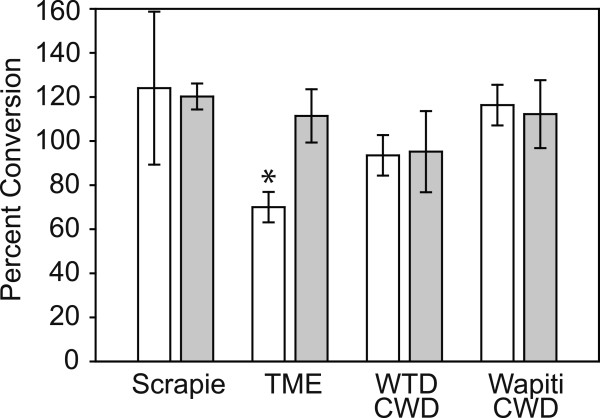
**Conversion efficiency ratios (CER) for bighorn sheep (BHS) and native hosts.** White bars indicate average CER for BHS substrates exposed to the indicated TSE agents and grey bars indicate average CER for native hosts. Error bars report standard deviations. Statistically significant differences between BHS and native host CER are indicated with an asterisk.

We calculated CER values by evaluating PrP^res^ levels in the pH 7.4 and 3.5 substrates for both the BHS and the native hosts for the TSEs we tested. We used data from at least three independently performed experiments and substrates prepared from three BHS, two domestic sheep, two mink and one white-tailed deer. Data for all of the average CER values +/− standard deviation are shown in Figure 6. We compared CER values for BHS to the normal hosts for the TSE using a Student’s *t*-tests and found that only TME in BHS was significantly different (t = 5.7517, df = 4, p = 0.0045).

### Protein misfolding cyclic amplification (PMCA)

As a control for our results from the CER assay, we performed a limited PMCA study to investigate misfolding of sheep PrP^C^ by PrP^TSE^ from CWD and TME. We subjected dilutions of classical scrapie, white-tailed deer CWD and TME in A^136^R^154^Q^171^ genotype domestic sheep PMCA substrate to cyclic sonication (Additional file 2). Following 48 h of PMCA, we detected PrP^res^ in samples seeded with either sheep scrapie or CWD, but not in samples seeded with TME. Substrate subjected to PMCA but lacking TSE agent seed did not contain detectable PrP^res^ and samples containing TSE agents that were not sonicated establish the background levels of PrP^res^.

## Discussion

Using the CER assay, we found evidence that this method recapitulates known species barriers of laboratory mice to TSEs and data to suggest that BHS could be susceptible to classical scrapie and CWD, and less susceptible to TME. Our present investigation and previous studies by others [23,24] suggest that the CER assay can be a valuable addition to other *in vitro* and *in vivo* measures of TSE species barriers such as cell-free conversions, PMCA and animal bioassays. Advantages of the CER assay include its low cost, short experimental timeframe and replacement of living animals with tissue samples (which need not be from transgenic mice or perfused). Additionally, the assay does not use radiation, reaction conditions are identical regardless of species or strain of TSE agent and, in our hands, the CER assay is robust and forgiving. Disadvantages of the assay include poor sensitivity compared to PMCA precluding the use of CER as a means to detect PrP^TSE^, the PrP^res^ product of conversion reactions is not known to be infectious and, importantly, the CER assay is less well-established than other methods of assessing species barriers which makes interpreting reductions in conversion ratios in the absence of other corroborating data difficult. For example, the correlation between a 50% CER and TSE transmission parameters (*e.g.* disease penetrance, length of incubation period) following experimental challenge remains undefined and further work is needed to characterize this assay for use in species where bioassay data are not available. Nonetheless, in our current study we did find a similar pattern of PrP^res^ formation when either CER or PMCA was used for conversion. Further studies comparing the two techniques is an interesting future direction.

We are not aware of any studies examining natural transmission of scrapie from domestic sheep to BHS, but in light of the sequence identity of BHS and domestic A^136^R^154^Q^171^ sheep prion proteins, we must consider scrapie a potential risk to BHS. Efforts to keep domestic sheep and BHS separated, as are prudent to prevent transmission of other pathogens from domestic sheep to BHS [30], are likely warranted around scrapie-infected farms. Further supporting the concept that BHS are at risk for acquiring scrapie is a report of the disease in mouflon (*Ovis orientalis*), another species of wild sheep [31].

In Figure 1, we show the overlap of BHS range with states and provinces known to have had scrapie cases since 2008. Reduced numbers of scrapie outbreaks in recent years, due to disease eradication efforts, may underrepresent the exposure of BHS to scrapie in years prior to 2008. Long-term environmental scrapie contamination may also still be contributing to BHS exposure to disease agent many years after scrapie outbreaks. If incubation periods of scrapie in BHS are greater than five years, exposure of BHS to pre-2008 scrapie flocks may only now have the potential to manifest as disease in BHS. The lack of current evidence for scrapie transmission to BHS could simply be due to insufficient surveillance, but other explanations, such as different susceptibilities by varying routes of exposure between domestic sheep and BHS or BHS being a “dead-end” host for scrapie, should also be explored. Sheep with *Prnp* genotype V^136^R^154^Q^171^ have been considered to be most -susceptible to classical scrapie and selective breeding efforts have focused on reducing the numbers of these animals in domestic sheep flocks across the U.S. as a scrapie risk reduction measure. Recent research, however, by Gonzalez *et al*. strongly suggests that *Prnp* genotype of the recipient sheep is not the sole factor determining its scrapie susceptibility *in vivo*[32]. In carrying out a series of codon 136 homologous, semi-homologous, and heterologous transmissions of two different natural scrapie isolates into domestic sheep, the study authors conclude that *Prnp* genotype alone cannot account for the diversity of disease phenotypes observed and that the “scrapie phenotype in sheep results from a complex interaction between source, donor and recipient factors” [32]. The susceptibility of BHS to scrapie is almost certainly dictated by this same interplay. More work is needed to explore the role of scrapie genetics on potential BHS disease transmission, as are analyses of BHS *Prnp* genetics using more geographically disparate samples.

The finding that white-tailed deer CWD agent could convert sheep PrP^C^ to PrP^res^ in either CER assays (Figure 6) or PMCA (Additional file 2) was notable given the sequence variations found between BHS or domestic sheep and white-tailed deer prion proteins (Figure 2), including serine to asparagine and asparagine to threonine changes in the “rigid loop” portion of the protein thought to control species susceptibility to CWD [33,34]. By CER assay, we similarly found conversion of BHS PrP^C^ by wapiti CWD heterozygous methionine/leucine at position 132. In wapiti, animals heterozygous or homozygous for leucine at prion protein amino acid 132 (L^132^) have a lengthened CWD incubation period [35] and L^132^ appears to limit CWD, but not classical scrapie, susceptibility in a transgenic mouse model [36]. Despite these amino acid mismatches, including those in the “rigid loop”, the CWD agents were still effective at misfolding PrP^C^ from BHS. In previous studies, we have also found that voles, which have mismatches in the “rigid loop” portion of the protein, are susceptible PrP misfolding and infection by cervid CWD [37,38].

Previous work on the species barrier of sheep to CWD has been equivocal. Using cell-free conversion assays, Raymond *et al.* found that A^136^Q^171^ domestic sheep PrP^C^ was not especially-well converted by CWD agent, but was the non-cervid substrate, among six tested species, that yielded the most PrP^res^[21]. In an animal study, Hamir *et al.* intracerebrally challenged eight domestic sheep of various *Prnp* genotypes with mule deer CWD [39]. One clinically-positive (heterozygous A/V^136^R^154^Q^171^) and one preclinical sheep (homozygous A^136^R^154^Q^171^) were identified at the conclusion of the study, indicating that sheep can be infected by CWD, although transmission is not especially facile.

The results of our conversion assays appear to be supported by *in vivo* work by Béringue *et al.* which indicate that V^136^R^154^Q^171^ ovinized transgenic mice challenged with wapiti CWD harbor high levels of splenic PrP^res^, indicating that sheep PrP^C^ is susceptible to misfolding by CWD agent [40]. At least one group has failed to observe clinical TSE signs in BHS when they were housed with a facility with CWD-infected animals [7]. Our results in combination with those of Béringue *et al.*, suggest that the lack of CWD transmission to BHS was not due to inability of BHS PrP^C^ to be misfolded by CWD agent, but must derive from other factors.

In our investigation, we used white-tailed deer and wapiti CWD, but have not yet investigated BHS prion protein conversion by mule deer CWD. Given the sequence similarity among cervid *Prnp* genes and our evidence that white-tailed deer and wapiti CWD can convert BHS PrP^C^ to PrP^res^, we expect CWD from the various species to behave similarly. In a previous report, Li *et al.* found less PrP^res^ generation in domestic sheep substrates when templated by wapiti CWD [23] than we found for BHS in our study. The genotype of the domestic sheep substrate in the previous study is unclear and differences between the sheep prion protein sequences or other species-specific differences could explain the limited conversion that they observed. Alternatively, differences in the genotypes of the wapiti CWD isolates used in the two studies could also explain variations in PrP^res^ levels in sheep substrates.

## Conclusion

The results from our study suggest that the CER assay has the potential to be a useful tool to screen TSE species barriers. Further comparisons with PMCA and bioassays will clarify the best uses of the assay and help to define CER that are < 100%. We found that BHS are unlikely to have resistance to domestic sheep classical scrapie due to their *Prnp* genotype. Our conversion reactions suggest that the species barrier protecting BHS from CWD may not be large and further studies, including *in vivo* experiments, are warranted. These animal challenge studies need not necessarily be performed in BHS, but could rather use *Prnp* genotype A^136^R^154^Q^171^ domestic sheep or existing transgenic mouse models [41]. Additionally, investigation into the susceptibility of BHS to atypical forms of scrapie is also an interesting future direction.

## Methods

### Materials

Chemicals and other reagents were of the highest quality possible and were from either Sigma-Aldrich (St. Louis, MO) or Fischer Scientific (Pittsburg, PA), unless otherwise specified.

### Tissues and disease agents

Animal work conducted at the National Wildlife Health Center was performed under institutional animal care and use committee protocol #EP080716. Brains from CD-1 Swiss mice intracerebrally-challenged with RML-strain TSE agent and Syrian hamsters intracerebrally-challenged with 263K TSE agent were used for control experiments. Healthy Swiss Webster mouse brains were purchased from Pel-Freez Biologicals (Rogers, AR) and healthy hamster brains were obtained from unchallenged animals.

Bighorn sheep brain tissues and retropharyngeal lymph nodes were collected from 28 animals of mixed gender and age from two herds culled in the state of Washington in 2010 by the United States Department of Agriculture [42]. Sheep brain was from a clinically-ill, classical scrapie positive, *Prnp* A^136^R^154^Q^171^ genotype (Genbank accession number: AAW88328.1) ewe maintained at the University of Idaho Caine Veterinary Teaching Center. Naïve control sheep brain was from *Prnp* A^136^R^154^Q^171^ genotype ewes from a certified scrapie-free facility acquired from Ovis Sheep (Sioux Falls, SD). Isolates of CWD from clinically-ill white-tailed deer (G^96^ genotype; Genbank accession number: AF156185.1) or wapiti (heterozygous M/L^132^ with other amino acids identical to Genbank accession number: ABS87888.1) were acquired from the Wisconsin Department of Natural Resources or the USGS Chronic Wasting Disease Positive Tissue Bank [43], respectively. Control white-tailed deer brain (G^96^ genotype) was from a hunter-harvested animal acquired in 2010 in Vilas County, Wisconsin. Brain from a transmissible mink encephalopathy (TME)-infected mink (*Neovision vision*) was from an animal experimentally infected with the agent and control mink tissue was the generous gift of Dr. Maria Shank (Wisconsin Veterinary Diagnostic Laboratory). All infected brain was homogenized in phosphate buffered saline (PBS) at 10% w/v using separate Dounce homogenizers and stored at −80°C until use. All control brains tested negative for PrP^res^ by immunoblot.

### Sequence analysis

Genomic DNA from BHS was isolated using a Genomic DNA Tissue Micro-Prep kit (Zymo Research, Irvine, CA) and was used to template PCR reactions using reagents from Marligen Biosciences (Rockville, MD) and the forward primer 5‘-TACGTGGGCATATGATGCTG-3‘ and the reverse primer 5‘-CTATCCTACTATGAGAAAAATGAG-3‘ specific to the extreme 5’ and 3’ regions of the coding sequence of the domestic sheep *Prnp* gene [21]. Sequencing of cervid *Prnp* genes was performed using published primers and protocols [44]. Amplicons were gel purified using the QIAquick gel extraction kit (Qiagen, Valencia, CA) and submitted to the University of Wisconsin Biotechnology Center for sequencing. Sequences were analyzed with Lasergene version 8.1.4 software (DNASTAR, Madison, WI). Multiple sequence alignments were performed using ClustalW2 software [45].

### Screening BHS tissue for preexisting TSEs

Tissue from the obex region of the medulla oblongata of BHS was homogenized in PBS (10% w/v) and subjected to PK digestion at 50 *μ*g∙ml^-1^ for 1 hr at 37°C and subsequently prepared for NuPAGE and immunoblotting. To test the sensitivity of our immunoblot, a dilution series of 10% classical scrapie brain homogenate was prepared in 10% BHS brain homogenate, PK digested at 50 *μ*g∙ml^-1^ for 1 hr at 37°C and prepared for NuPAGE and immunoblotting. Retropharyngeal lymph nodes from BHS were analyzed at the Wisconsin Veterinary Diagnostic Laboratory using the TeSeE ELISA kit and standard procedures (BioRad, Hercules, CA). Brains from BHS used for conversion efficiency ratio assay substrates were also screened using PMCA as described in the PMCA section.

### Conversion efficiency ratio (CER) assay substrate preparation

Uninfected brain homogenate used in the *in vitro* conversion assay was prepared using the method described in Zou *et al*. [24]. Briefly, brain tissue was homogenized at 10% w/v in cell lysis buffer (100 mM NaCl, 10 mM EDTA, 0.5% NP-40, 0.5% deoxycholate, 10 mM Tris pH 7.5). Guanidine HCl (3 M) in PBS at either pH 3.5 or 7.4 was added to the homogenate at a 1:1 ratio and gently rotated at room temperature for 5 hr. Protein was then precipitated with 4 volumes of methanol at −20°C for 16–18 hr and sedimented by centrifugation at 13,000 *g* for 30 min at 4°C. Pellets were resuspended in conversion buffer (0.05% SDS, 0.5% Triton X-100 in PBS at pH 7.4), briefly sonicated in a cuphorn sonicator, and the resultant conversion assay substrate was stored at −80°C until use.

### Conversion efficiency reactions

Conversion reactions were performed in 200 *μ*l MAXYMum Recovery low binding PCR Tubes (Axygen Scientific, Union City, CA) by adding 5 *μ*l of 10% infected brain homogenate to 95 *μ*l of uninfected brain substrate that was previously denatured at either pH 3.5 or 7.4 as described earlier. Control samples lacking brain substrate contained only TSE agent and conversion buffer. Tubes were vortexed briefly then placed in a 96-well PCR tube thermo-shaker (Hangzhou All Sheng Instruments, Hangzhou City, Zhejiang Province, China) for 24 hr at 37°C at 1000 rpm. After shaking, reactions were treated with sarkosyl to a final concentration of 2% w/v and digested with PK (100 *μ*g∙ml^-1^) for 1 hr at 37°C. We found that adding sarkosyl to samples post-incubation aided PK digestion of prion protein in unseeded substrates (data not presented). Samples were then prepared for NuPAGE and immunoblotting. Assays with BHS substrate were performed using at least two individual animals and all assays were individually replicated at least three times.

### Protein misfolding cyclic amplification (PMCA) 

Procedures for PMCA were based on those previously published for domestic sheep [46]. Briefly, PMCA substrates were prepared from uninfected A^136^R^154^Q^171^ genotype domestic sheep or BHS medullas homogenized to 10% w/v using Dounce homogenizers in ice-cold conversion buffer composed of PBS with 150 mM NaCl, 1% Triton X-100, 4 mM EDTA and mini-Complete protease inhibitor (Roche, Indianapolis, IN) at pH 7.4. Classical scrapie, white-tailed deer CWD or TME agents were diluted into the domestic sheep substrate at dilution factors of 10^-2^ or 10^-3^ of a 10% w/v brain homogenate. Substrate from BHS was left unseeded to test for pre-existing PrP^Sc^. Samples were either frozen at −80°C or immediately subjected to PMCA with bead enhancement [47]. Samples for PMCA were sonicated for 48 h at 37°C using cycles composed of a pulse at 80% amplitude for 20 s followed by 30 min of incubation. Samples for serial PMCA were reseeded into fresh BHS substrate at a 1:10 ratio. After PMCA, some samples were digested with PK (100 *μ*g∙ml^-1^) for 1 hr at 37°C prior to immunoblotting. Experiments were independently replicated twice and negative control samples lacking TSE agents were subjected to PMCA and were included in each experiment.

### NuPAGE and immunoblotting

Prior to NuPAGE, lithium dodecyl sulfate sample buffer and NuPAGE reducing agent (both from Invitrogen, Carlsbad, CA) were added to samples to a final concentration of 1×. Samples were then heated at 95°C in a dry block heater for 5 min, cooled and loaded into 12% Bis-Tris NuPAGE gels. Proteins were transferred to polyvinyldifluoride membranes (Immobilon-P, Millipore, Billerica, MA), blocked in non-fat dry milk and immunoblotted using SAF 83 or BAR 224 monoclonal antibodies (Cayman Chemical, Ann Arbor, MI) at a 1:5,000 dilution and polyclonal goat anti-mouse IgG (Santa Cruz Biotechnology, Santa Cruz, CA) at a 1:10,000 dilution. Immunoreactivity was detected using SuperSignal West Pico chemiluminescent system (Thermo Scientific Pierce Chemical, Rockford, IL) and an EC3 imaging system (UVP, Upland, CA). For presentation purposes, some irrelevant lanes were excised from images of membranes and no further changes to brightness or contrast were made following excision. Data from separate gels presented in the same figure are presented in separate boxes.

### Calculation of conversion efficiency ratio (CER)

Substrates at pH 7.4 and 3.5, which were prepared at the same time from the same brain homogenate starting material, were incubated and shaken with a TSE agent, PK treated, ran on the same gel and immunoblotted together. Levels of PrP^res^ were measured by densitometry using the VisionWorks LS software on the EC3 imaging system. The density of the pH 7.4 sample was divided by the density of the pH 3.5 sample and multiplied by 100 to produce a percentage. Data from replicates of experiment were compiled to generate a mean CER for a species with a given TSE agent ± standard deviation. An analysis of variance was performed using the Tukey-Kramer minimum significance differences method and Student’s *t* test performed with GraphPad software (La Jolla, CA).

## Abbreviations

TSE: Transmissible spongiform encephalopathy; CWD: Chronic wasting disease; BHS: Bighorn sheep (*Ovis canadensis*); Prnp: Gene encoding the prion protein; PrPC: Normal cellular prion protein; PrPTSE: Disease-associated prion protein; PK: Proteinase K; PrPres: Proteinase K-resistant prion protein; PMCA: Protein misfolding cyclic amplification; CER: Conversion efficiency ratio; TME: Transmissible mink encephalopathy; PBS: Phosphate buffered saline.

## Competing interests

The authors declare that they have no competing interests.

## Authors’ contributions

ARM, CMC and HC performed experiments, analyzed results and helped draft the manuscript. CJJ conceived of the study, performed experiments, analyzed results and helped draft the manuscript. All authors have read and approved the final version of the manuscript.

## Supplementary Material

Additional file 1 **Bighorn sheep (BHS) and native host conversion efficiency ratios substrates.** Substrates prepared at either pH 7.4 or 3.5 from BHS, mink, domestic sheep or white-tailed deer (WTD) were shaken in the absence of TSE agents. No proteinase K-resistant prion protein was found by immunoblot with monoclonal antibody BAR 224. Click here for file

Additional file 2 **Protein misfolding cyclic amplification (PMCA) using domestic sheep (A**^**136**^**R**^**154**^**Q**^**171**^**) genotype substrate.** The indicated TSE agents were diluted to 10^-2^ or 10^3^ from 10% w/v stocks of brain homogenate into domestic sheep PMCA substrate and subjected to 96 cycles of sonication. Proteinase K (PK)-resistant prion protein levels were assessed by immunoblotting with monoclonal antibody BAR 224. As a control, substrate without TSE agent seed was subjected to PMCA cycling. Samples of sheep substrate containing the indicated TSE agents, but not subjected to PMCA, served to establish background levels of PK-resistant prion protein Click here for file
