# Supplementary material for: In vitro prion protein conversion suggests risk of bighorn sheep (Ovis canadensis) to transmissible spongiform encephalopathies
Source: BMC Vet Res. 2013 Aug 9;9:157. doi: 10.1186/1746-6148-9-157 (PMC3751320; doi:10.1186/1746-6148-9-157)
Supplement: Additional file 1 — Bighorn sheep (BHS) and native host conversion efficiency ratios substrates. Substrates prepared at either pH 7.4 or 3.5 from BHS, mink, domestic sheep or white-tailed deer (WTD) were shaken in the absence of TSE agents. No proteinase K-resistant prion protein was found by immunoblot with monoclonal antibody BAR 224. [file 1746-6148-9-157-S1.pdf]

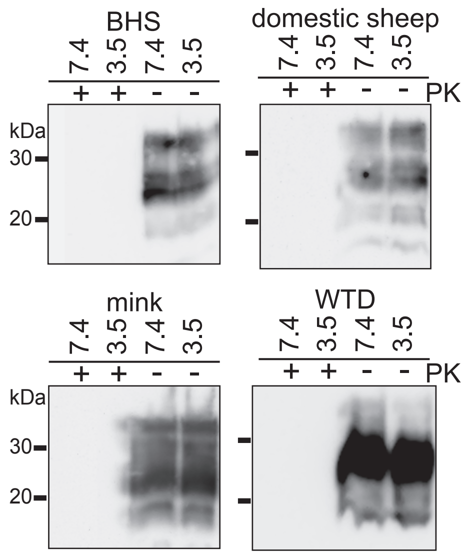

Additional Figure 1. Bighorn sheep (BHS) and native host conversion efficiency ratios substrates. Substrates prepared at either pH 7.4 or 3.5 from BHS, mink, domestic sheep or white-tailed deer (WTD) were shaken in the absence of TSE agents. No proteinase K-resistant prion protein was found by immunoblot with monoclonal antibody BAR 224.

Additional Figure 1
