# Supplementary material for: In vitro prion protein conversion suggests risk of bighorn sheep (Ovis canadensis) to transmissible spongiform encephalopathies
Source: BMC Vet Res. 2013 Aug 9;9:157. doi: 10.1186/1746-6148-9-157 (PMC3751320; doi:10.1186/1746-6148-9-157)
Supplement: Additional file 2 — Protein misfolding cyclic amplification (PMCA) using domestic sheep (A136R154Q171) genotype substrate. The indicated TSE agents were diluted to 10-2 or 103 from 10% w/v stocks of brain homogenate into domestic sheep PMCA substrate and subjected to 96 cycles of sonication. Proteinase K (PK)-resistant prion protein levels were assessed by immunoblotting with monoclonal antibody BAR 224. As a control, substrate without TSE agent seed was subjected to PMCA cycling. Samples of sheep substrate containing the indicated TSE agents, but not subjected to PMCA, served to establish background levels of PK-resistant prion protein [file 1746-6148-9-157-S2.pdf]

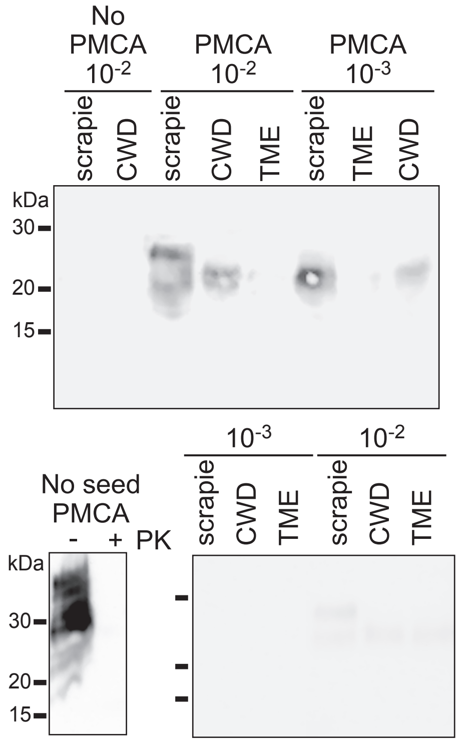

Additional Figure 2. Protein misfolding cyclic amplification (PMCA) using domestic sheep ($A^{136}R^{154}Q^{171}$) genotype substrate. The indicated TSE agents were diluted to 10^{-2} or 10^{-3} from 10% w/v stocks of brain homogenate into domestic sheep PMCA substrate and subjected to 96 cycles of sonication. Proteinase K (PK)-resistant prion protein levels were assessed by immunoblotting with monoclonal antibody BAR 224. As a control, substrate without TSE agent seed was subjected to PMCA cycling. Samples of sheep substrate containing the indicated TSE agents, but not subjected to PMCA, served to establish background levels of PK-resistant prion protein.
